# Scoping Review and Bibliometric Analysis of the Term “Planetary Health” in the Peer-Reviewed Literature

**DOI:** 10.3389/fpubh.2020.00343

**Published:** 2020-07-29

**Authors:** Verena Rossa-Roccor, Emily Sohanna Acheson, Federico Andrade-Rivas, Michelle Coombe, Saori Ogura, Laura Super, Andy Hong

**Affiliations:** ^1^School of Population and Public Health, University of British Columbia, Vancouver, BC, Canada; ^2^Department of Geography, University of British Columbia, Vancouver, BC, Canada; ^3^Vicerrectoría de Investigaciones, Universidad El Bosque, Bogotá, Colombia; ^4^Department of Occupational Science and Occupational Therapy, University of British Columbia, Vancouver, BC, Canada; ^5^Department of Forest and Conservation Sciences, University of British Columbia, Vancouver, BC, Canada; ^6^The George Institute for Global Health, University of Oxford, Oxford, United Kingdom; ^7^Department of City and Metropolitan Planning, University of Utah, Salt Lake City, UT, United States

**Keywords:** planetary health themes, holistic health fields, interdisciplinary research, planetary health collaborations, keyword network analysis

## Abstract

**Background:** Planetary health is an emerging holistic health field to foster interdisciplinary collaborations, integrate Indigenous knowledge, facilitate education, and drive public and policy engagement. To understand to what extent the field has successfully met these goals, we conducted a scoping review and bibliometric analysis.

**Methods:** We searched 15 databases from 2005 to 2019 for peer-reviewed publications with the term “planetary health” in the title, abstract and/or keywords, with no language or geographical location limitations. We classified results into four categories (commentaries, comprehensive syntheses, educational material, and original research) and categorized original research according to expert-derived planetary health themes. Our bibliometric analysis highlighted publications over time, collaborations, and networks of keywords.

**Findings:** Only 8.1% (*n* = 22) were research articles. Publications rose rapidly from 8 to 64 publications per year in 2015–2018. The top five author affiliation countries for most publications were the US, UK, Australia, Canada, and New Zealand, and the top five collaborations were a subset of pairwise combinations between the US, UK, Australia, and Canada. The most common author keywords were the following: planetary health, climate change, ecology, and non-communicable diseases. Keyword co-occurrences clustered around high-level concepts (e.g., Anthropocene) and food system-related topics; two clusters lacked a theme.

**Interpretation:** We show that the term planetary health is used mainly in commentary-like publications, not original research. Additionally, more global collaborations are lacking. Interdisciplinary work, as represented by keyword co-occurrence networks, is developing but could potentially be extended. The planetary health community should promote more worldwide research and interdisciplinary collaborations.

## Introduction

We are in a time of environmental crisis primarily generated through human activity. Humans have driven climate change (1), increased desertification (2), altered geochemical cycling (3), increased ocean acidification (4), and contributed to the sixth mass extinction event on Earth (5). Our health and survival depend on the environment for “ecosystem services,” which are non-economic benefits provided by healthy ecosystems such as food production, clean water, protection from natural hazards, and spiritual fulfillment (6). However, conventional public health practices have not yet fully integrated the health of the ecosystems into the assessment of global public health trends but, instead, have largely focused on quantitative assessments of human life expectancy and disease status (e.g., disability-adjusted life-years, healthy life expectancy) (7). When comparing human health and environmental trends, disturbing patterns emerge where conventional public health successes come at a cost to environmental health (8).

Conversely, since the 1970s, we have seen the emergence of a number of holistic disciplines such as ecosystem health, conservation medicine, EcoHealth, One Health, GeoHealth, and planetary health which aim to bridge the false dichotomy between human health and ecological sustainability (9). These fields vary in their historical context and specific emphasis (9, 10). The planetary health field is one of the youngest holistic fields and looks principally through the lens of human health; it defines itself as “the achievement of the highest attainable standard of health, well-being, and equity worldwide through judicious attention to the human systems—political, economic, and social—that shape the future of humanity and the Earth's natural systems that define the safe environmental limits within which humanity can flourish.” (8).

If planetary health intends to serve as an interdisciplinary platform for global change, it is necessary to understand who chooses to self-identify with the term “planetary health” and provide a critical view of the emerging concepts, visions, and research interests of the researchers who are embracing it. Lessons learned from planetary health research must be spread widely; however, currently even transdisciplinary health research tends to be published—and therefore isolate its conclusions—within specific academic fields (9). In order to properly evaluate the field of planetary health and its potential impact, it is critical to have a published, replicable baseline that can be used as a metric now and in the future. Our study situates the current influence of planetary health by exploring how the term has been used in the peer-reviewed literature across academic disciplines. Our specific aims are to understand the following:

- the key content themes that receive most attention in peer-reviewed planetary health research,
- by whom and in what regions the term planetary health is used, and to what degree interdisciplinary and international collaborations exist, and
- what the resulting knowledge gaps for self-identified planetary health work are.

We conclude with recommendations for growing the field of planetary health and acceptance that the health and well-being of humankind need rapid, decisive action to safeguard the health of the planet.

## Methods

### Literature Review

#### Data Sources

We searched electronic databases for articles from a variety of fields beyond the health sciences. The sources and inclusion and exclusion criteria are provided in Table 1. We used Preferred Reporting Items for Systematic Reviews and Meta-Analyses (PRISMA) to document the search process and results (11).

**Table 1:** Data sources and criteria of the literature search used for the scoping review and bibliometric analysis of the planetary health field.

**Data source or criteria type**	**Restrictions**
Electronic database sources used for literature search	ABI/Inform, Agricultural and Environmental Science Database (AESD), Bibliography of Native North Americans, CAB Direct and Global Health, EBSCOhost, Geobase, Informit Indigenous Collection, iportal USASK, JSTOR, MEDLINE Ovid, Native Health Database, PAIS Index, ProQuest Dissertations and Thesis Global, PsycINFO, and Web of Science
Search terms	“Planetary Health” OR “Planetary health” OR “planetary health” in the title, abstract and/or keywords
Search restrictions	Results between January 1, 2005 and October 21, 2019 (the day of the literature search) Results were peer-reviewed literature No restrictions placed on language or geographic location
Exclusion criteria	Publications that did not use the term planetary health as defined by the Planetary Health Alliance: “Planetary health is a field focused on characterizing the human health impacts of human-caused disruptions of Earth's natural systems.” Publications not accessible in their entirety (e.g., if only a citation or abstract to a full text was available) Secondary sources (e.g., book or movie reviews, and errata)

#### Screening and Decision Making

Each author searched a subset of databases. We exported all resulting citations and removed duplicates manually in a bibliographical database manager (Zotero 5.0.58). After duplicate removal, each author independently reviewed the remaining articles with inclusion criteria; articles meeting the exclusion criteria were removed. If any decisions diverged, the group discussed results until a consensus was reached.

#### Content Analysis

We first reviewed articles to classify each as one of the following: (i) a commentary, opinion, editorial, or letter to the editor; (ii) an education-content description (i.e., providing educators or administrators with instructional ideas or issues in a given discipline); (iii) a synthesis, literature review, or framework; or (iv) original research. We then conducted a content analysis (12) which allowed us to categorize the original research articles according to the 14 expert-derived themes in planetary health (13), where more than one theme per article could be chosen. These themes included nine areas that describe environmental changes (water scarcity, changing food systems, urbanization, biodiversity shifts, natural disasters, climate change, changing land use, and land cover, global pollution, and changing biogeochemical flows) as well as five areas of health impacts (non-communicable diseases, infectious diseases, mental health, nutrition, civil strife, and displacement). We used these themes as a guiding framework for our scoping review, not as an exhaustive list. In addition, our analysis was informed by the cross-cutting principles for planetary health education (14) and other planetary health literature (9, 15, 16) that have emphasized the importance of cultural identity, education, governance, and policy for the field. Given that these topics may not be captured as main content themes in original research manuscripts, we analyzed all articles for content on Indigenous knowledge, planetary health education, and/or evaluation of—or concrete recommendations for—policies. We conducted the classification and content analysis in a blinded, iterative process, which meant that each article was read by at least two authors on separate occasions to increase replicability of the results.

### Bibliometric Analysis

#### Publications Over Time and Country Affiliations

To understand temporal and spatial distribution of the term planetary health in the peer-reviewed literature, we analyzed the distributions of annual scientific production per year. We explored which countries were affiliated with planetary health publications by identifying the unique countries affiliated with authors of each publication. For example, if a publication has five authors from Canada and five authors from Kenya, Canada and Kenya would each be recorded once. We determined country affiliations by author address at time of the publication and mapped all possible pairings, using ArcMap version 10.5.1 (17).

#### Network Analysis of Keyword Co-occurrences

To further examine the emerging interdisciplinary research clusters and collaborations within the field, we conducted a bibliometric analysis using a subset of articles that included author-provided keywords in the manuscript. Following previously reported methods (18, 19), we used a similarity measure to construct a network with keywords as nodes and keyword co-occurrences as relations. Keywords were made consistent by unifying plural/singular forms, spelling out acronyms, and unifying hyphenated/non-hyphenated terms. Given that planetary health is an emergent field with little consensus on how concepts are applied within the field, we did not merge potentially similar keywords. We conducted a sensitivity analysis by first exploring different samples of keywords ranked by their degree (i.e., number of connections) in terms of the possibility to discern clusters and the quantity of nodes in the output. This sensitivity analysis confirmed that 40 was the smallest number of plotted keyword connections that best represented the current thematic structure of the planetary health field at a reasonable breadth and depth. Second, we relied on previously developed criteria to choose the most appropriate clustering method that provided the best visual representation of the keyword network (Louvain method) (20). All analyses were done using R v.3.6.0 and the R bibliometrix package v.2.3.1 (18).

## Results

### Literature Search Results

**Figure 1:**
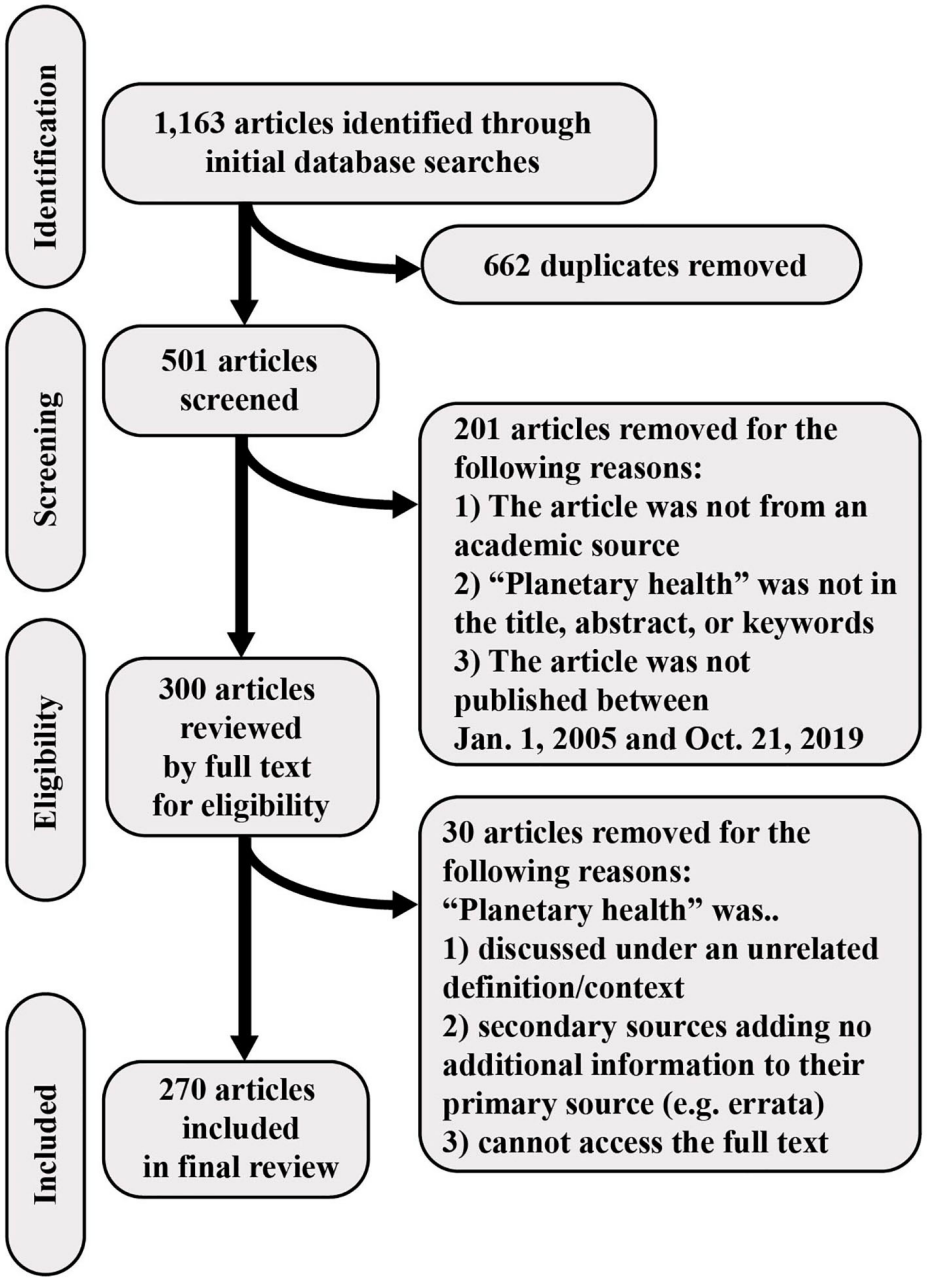
PRISMA flow chart detailing the review process and selection of final sample of publications.

The initial literature search yielded a total of 1,163 publications. After removing duplicates and screening for inclusion and exclusion criteria, we retained 270 articles in the final list of publications used for analysis of content, country affiliations, and publications over time (Figure 1). It is important to note the skewed distribution of publications across journals, with a few journals being the source of a large number of articles and the majority of journals each having published only one of the articles included in the analysis. The leading journals by number of publications are the following: The Lancet Planetary Health with 42 publications, The Lancet with 31 publications, Challenges with 17 publications (16 of which were published in the topical collection “the emerging concept of planetary health”) (21) and Public Health Reviews with six publications. We provide a full list of references of the final results in the Supplementary Material (Supplementary Table 1).

#### Content Analysis

We found that 66.3% (*n* = 179) of articles that met our inclusion criteria could be categorized as opinion pieces such as commentaries, letters to the editor, or calls to action; 23.3% (*n* = 63) were categorized as comprehensive syntheses, literature reviews, or framework suggestions; 2.6% (*n* = 7) described educational content such as planetary health courses; and 8.1% (*n* = 22) reported original research. Indigenous knowledge and traditions were considered in 7.4% (*n* = 20) of the articles; an evaluation of or concrete recommendations for policies were made in 10.4% (*n* = 28) of our final list of articles. The 22 original research articles matched expert-derived themes put forward by the Planetary Health Alliance (PHA) as depicted in Table 2. The most commonly discussed theme in terms of environmental changes was climate change (*n* = 5); for health impact, nutrition was most often the theme of the research (*n* = 10). Natural disasters and civil strife and displacement yielded no results in our review. Five publications that were deemed original research did not fit within the 14 themes.

**Table 2:** Number of original research publications by planetary health theme.

**Theme**	Number of publications^*^
**Environmental changes**	
Climate change	5
Biodiversity shifts	2
Changing food systems	2
Changing land use and land cover	2
Global pollution	2
Changing biochemical flows	1
Urbanization	1
Water scarcity	1
Natural disasters	0
**Health impacts**	
Nutrition	10
Infectious diseases	1
Mental health	1
Non-communicable diseases	1
Civil strife and displacement	0

* *More than one theme per article could be chosen*.

### Bibliometric Analysis Results

#### Publications Over Time and Country Affiliations

**Figure 2:**
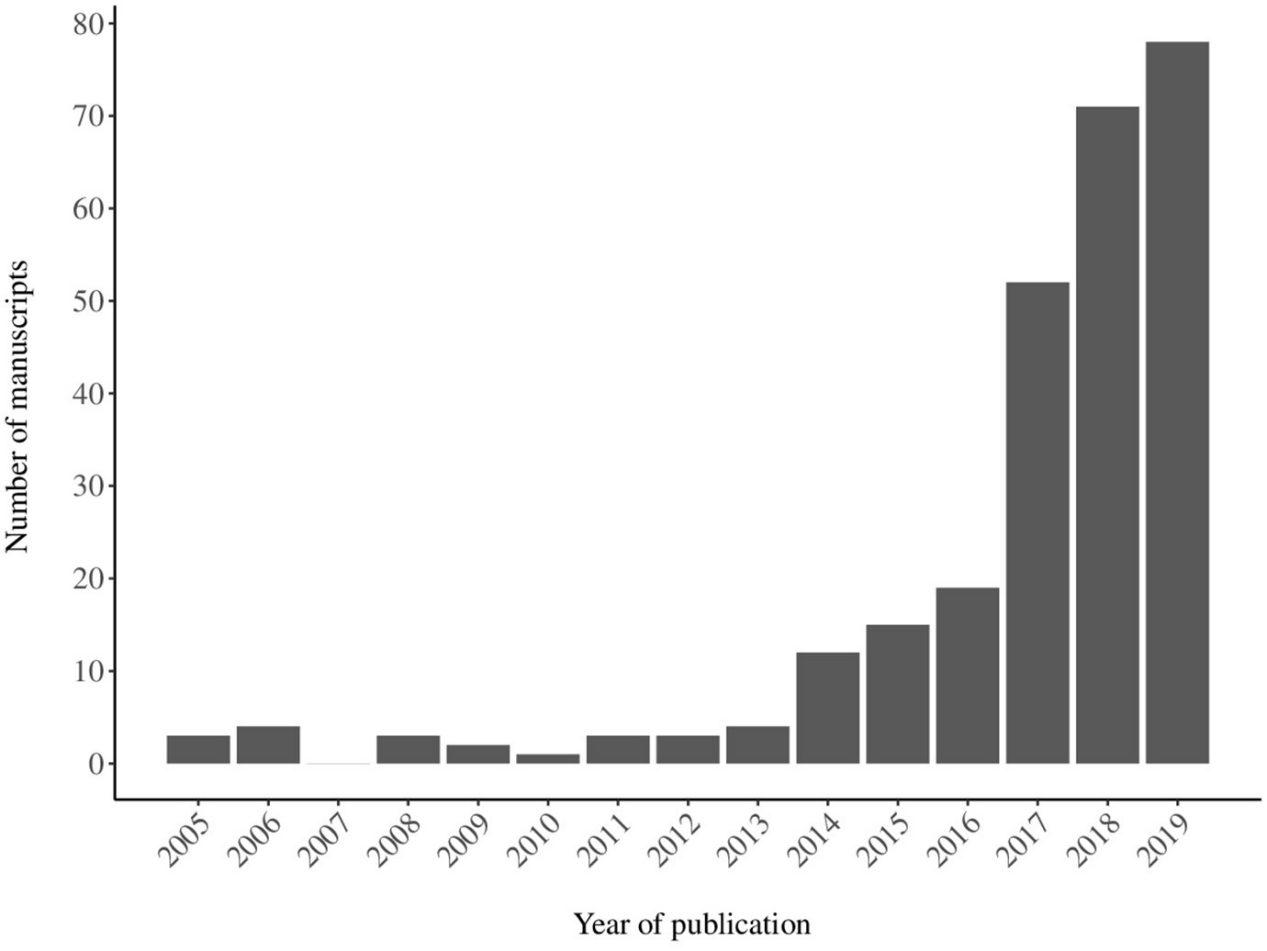
The number of self-identified planetary health manuscripts published by year based on 270 articles included in scoping review.

We used the full set of search results (*n* = 270) for the assessment of country affiliations and publications over time. Publications have increased noticeably since 2016, with 17 publications in 2016 compared to eight publications in the previous year and continued to rise until reaching 64 publications in 2018 (Figure 2). This increasing trend is consistent with the date of publication of the report of the Rockefeller Foundation-Lancet Commission on planetary health in 2015 (8) and the inaugural meeting of the PHA in 2016.

**Figure 3:**
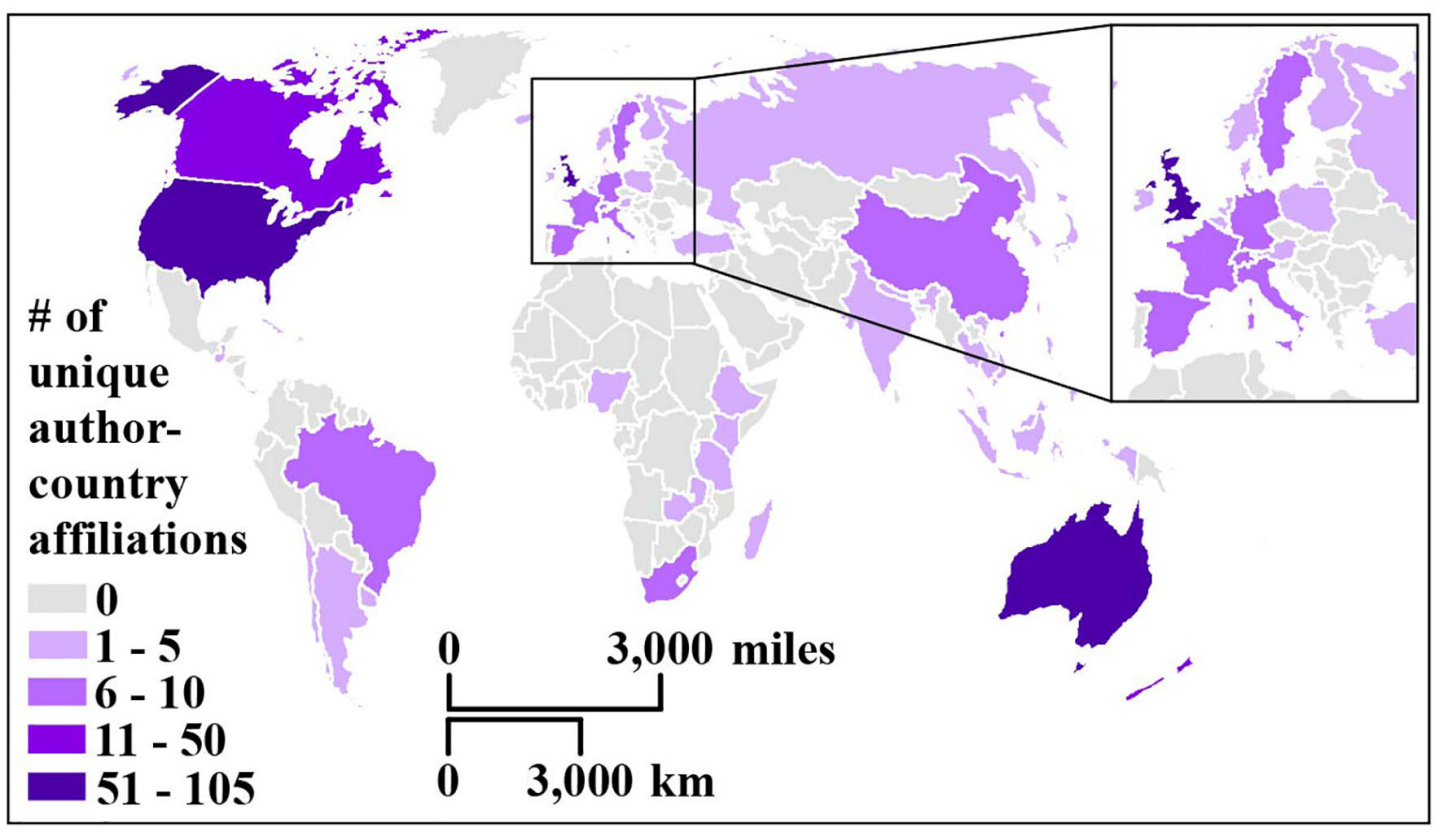
Map of the number of author-country affiliations based on the unique country affiliations of all coauthors per publication. Inset map shows the United Kingdom, Ireland, and part of Northern Europe.

A total of 49 unique countries contributed to the 270 reviewed articles. An analysis of the 447 author-country affiliations revealed that the majority of publications stemmed from Anglophone countries (Figure 3). The top five author affiliation countries with the most publications were the following (out of *n* = 447): United States (*n* = 105), United Kingdom (*n* = 95), Australia (*n* = 57), Canada (*n* = 34), and New Zealand (*n* = 11). These affiliations were calculated based on the number of unique country affiliations across all authors per publication.

There were 346 pairs of collaborating countries and a total of 186 unique pairs (Supplementary Figure 1). The top five country collaborations were the following (out of *n* = 346 pairs): United Kingdom and the United States (*n* = 20), Australia and the United States (*n* = 19), Australia and the United Kingdom (*n* = 13), Canada and the United States (*n* = 10), and Australia and Canada (*n* = 7).

#### Network Analysis of Keyword Co-occurrences

**Figure 4:**
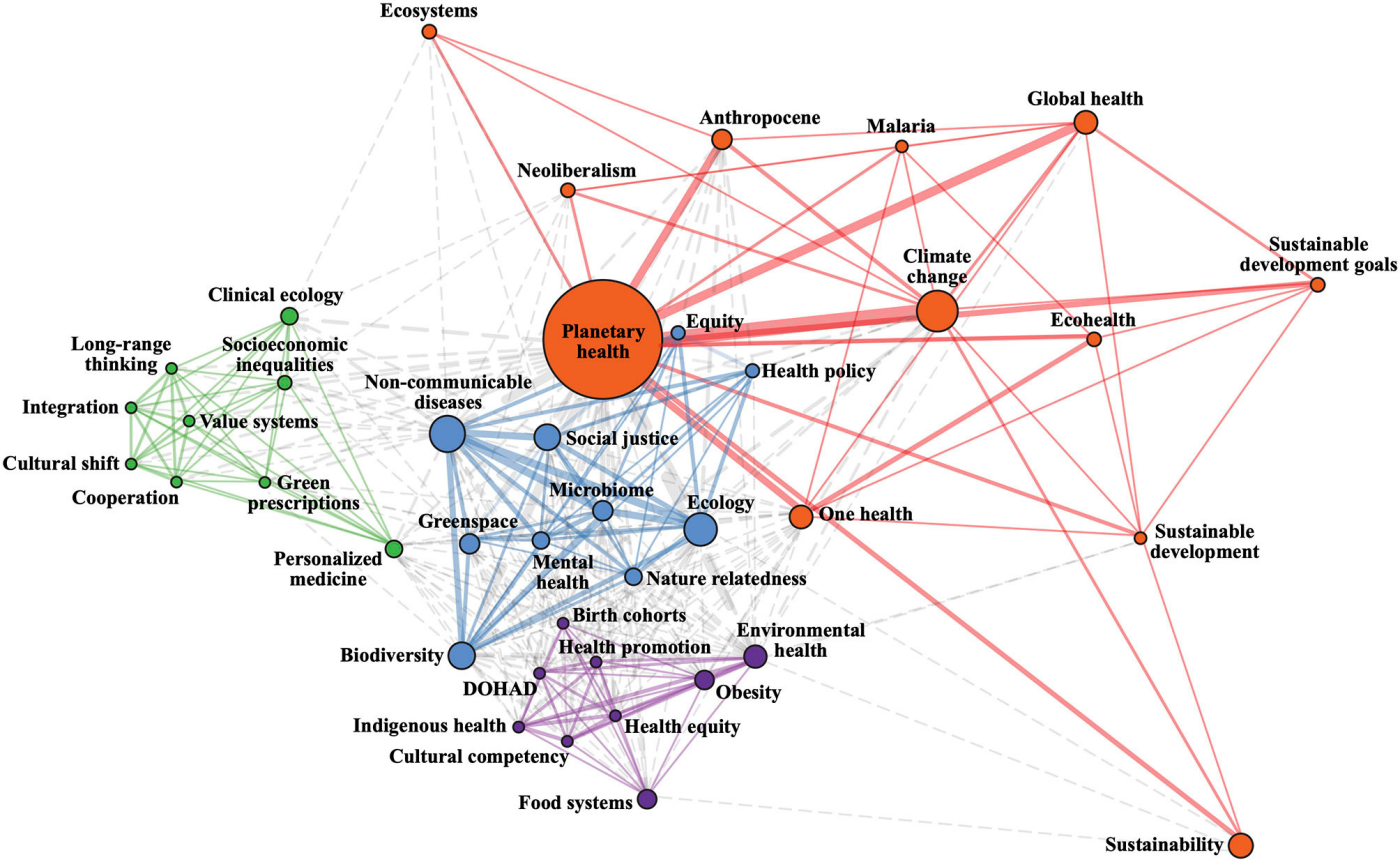
Network plot representing keyword clusters. Colored lines represent connections between keywords within the same cluster while dotted lines represent connections between keywords in different clusters. Increasing line thickness and node size represent higher numbers of connections between clusters and higher usage of a keyword, respectively.

We used a smaller subset of *n* = 103 articles for the network analysis of keyword co-occurrences because *n* = 167 articles did not provide author-derived keywords. The majority (*n* = 129, 77.2%) of the 167 articles without author-provided keywords were opinion pieces. The network plot (Figure 4) provides a visual representation of the most prevalent keywords within the planetary health field and how they are connected to each other using keyword frequency and co-occurrences.

The size of the node represents the frequency of keywords, the width of the line between keywords represents the number of co-occurrences, and the different colors correspond to identified clusters of keywords. The largest nodes are the keywords used by more than ten manuscripts: planetary health, climate change, ecology, and non-communicable diseases.

Four clusters were identified (red, blue, purple, and green). Keywords in the red cluster are mostly related to high level concepts (e.g., Anthropocene, sustainability, neoliberalism) and holistic research fields (i.e., planetary health, global health, One Health, EcoHealth). The strongest connections within this cluster are planetary health-climate change and planetary health-global health. The blue cluster had several keywords with a high level of connectivity and frequency, but no clear main keyword dominating the cluster. Non-communicable diseases, ecology, social justice, biodiversity, and microbiome are closely related in this cluster, but other emerging topics in planetary health can also be elucidated. For example, the growing research exploring the connection between mental health and environmental factors (e.g., green space and biodiversity) is evident. The purple cluster shows environmental health, obesity, and food systems as the most frequent and connected keywords. It is worth noting that Indigenous health is the only keyword in this network referring to a specific human population, and it is linked closely with obesity, environmental health, and food systems. The green cluster does not have a visible central keyword. Concepts like value systems, cultural shift, integration, cooperation, and long-range thinking suggest that this cluster of keywords refers to discussions of the underlying paradigms associated with planetary health. Keywords within the green cluster come mainly from publications that form a topical collection in planetary health (21), suggesting a particular vision of the field represented in this journal.

## Discussion

### Interpretation

Planetary health is an emerging holistic field that is rapidly growing. Our work provides a new baseline analysis of the spread of planetary health as a research topic in the peer-reviewed literature. Planetary health as a new field aims to secure a sustainable future through providing cutting-edge scientific evidence, ensuring global equity in health, building a transdisciplinary community, training of the next generation, and engaging in public outreach and policy making (9, 10, 13, 14). In addition, some authors have also highlighted the central role of values, advocacy, movement building, knowledge integration, and solution-based approaches (14, 16).

Our research shows that the term planetary health is currently used overwhelmingly in opinion pieces that convey the necessity of adopting a holistic health viewpoint to achieve bold political action and guarantee the well-being of humans and our planet. In contrast, only a small percentage of authors currently use the term planetary health when publishing original research. This arguably stands in clear contrast to the actual amount of research being conducted within the themes that have been identified as pertinent to planetary health. For example, when looking at the references informing the Rockefeller Foundation-Lancet Commission Report on Planetary Health (8), only two out of 432 cited articles use the term planetary health in the title, abstract or keywords, meaning that the majority of work in this field remains scattered across different disciplines.

The lack of the use of the term planetary health in research has many possible reasons. First, planetary health is a new term. Although it first emerged in the environmental movement of the 1970s and 1980s, it only found its way into the focus of academic discourse around 2015 after the publication of the Rockefeller Foundation-Lancet Commission Report on Planetary Health (8). This is reflected in the results of our bibliometric analysis which shows that the most significant rise in publications using the term planetary health began around 2015. The predominance of publications in only two journals—namely the Lancet Planetary Health and the Lancet—may also be a reflection of the relative youth of the planetary health field. By comparison, the One Health field has shown a doubling of publication rate approximately every 5 years since 1990 (22). Indeed, although the origins of planetary health are only in 2015, our results show a similar, sharp increase in publications over time. However, the minimal diversity in journals could also indicate the formation of a new “planetary health” disciplinary silo (vs. true transdisciplinary work) or that authors may not choose to ascribe the planetary health keyword to their work when publishing in other journals. Second, based on conversations with researchers attending planetary health conferences, we learned that some researchers are critical of the term and reluctant to use it to describe their work for reasons such as the perception that the term is not intuitive, that it does not sufficiently reflect schools of thought outside of the health sciences, or that it leads to misunderstandings about the research aims. Given that advocacy is a central tenet of the planetary health field under which a positive relationship between environmental and human health is assumed, some researchers have also raised their concern that the “planetary health label” may indicate the potential for perceived biased scientific results. Conversely, other colleagues who intentionally use the term planetary health have argued that, given the scope and urgency of the issue of global environmental degradation and its potentially devastating effects on human health and well-being, the self-image of scientists needs to embrace the role of being advocates. This can be seen in other holistic health fields (e.g., One Health, EcoHealth) which also value public outreach (23, 24) and have continued to grow and increased their impact despite the apparent tension between scientific neutrality and advocating for evidence-based political change.

Many colleagues have also mentioned their efforts to highlight their research as planetary health at every opportunity to reach a broad audience and to break down disciplinary barriers. Indeed, integration of different disciplines under the planetary health umbrella seems to be emerging. The keyword co-occurrences show that clusters are not conceptually distinct from each other and that traditionally isolated concepts are being linked together (e.g., social justice and microbiome, mental health and green spaces/urban planning). Unfortunately, inter- and transdisciplinary work remains difficult. Although efforts are being made to create research hubs and centers for excellence and interdisciplinary institutes (25), academic research still is largely being conducted in disciplinary silos (25). The trend to work and cite within a given discipline occurs even in other holistic, transdisciplinary health fields such as One Health, emphasizing the many barriers to transdisciplinary work (22). Overcoming traditional silos often takes more time, flexibility, and openness than traditional approaches and requires strong leadership in particular areas such as in emphasizing teamwork, opportunities for communication, shared terminologies, and specific funding for transdisciplinary projects (26). The integration of traditionally disparate topics (e.g., non-communicable disease, ecology, social justice) is a promising move toward ongoing transdisciplinary work in the planetary health field. However, researchers and funders could greatly benefit from emphasizing such approaches to continue building novel and integrative research across countries, research groups, and disciplines.

The paucity of original research with the term planetary health is also reflected in the literature gaps within the 14 themes that we used as a guiding framework. For example, natural disasters and civil strife and displacement yielded no original research results in our review. The latter has a vast body of literature, such as in the political science field (27), but has only recently crossed over into the health field. The planetary health community could benefit from expanding beyond the health and environmental sciences and better incorporate insights from the social sciences. More specifically, this may mean an expansion of these 14 themes. Five of the original research articles that we found did not fit within these themes but suggested new, primarily qualitative themes: inter- and trans-disciplinary research development and directions (28, 29), ecological behavior and spiritual healing (30–32) and participatory methods of communication. The need for an expansion of the themes was supported by the lack of congruence between the keywords provided by authors and the expert-derived themes used in our content analysis.

Looking beyond the health and environmental sciences might also be helpful in identifying effective strategies to prompt policy change. In our study, 10.4% (*n* = 28) of the articles addressed pathways to policy change or analyzed existing policies that went beyond mere calls to action. An invitation and extension of the planetary health field to encompass knowledge translation scholarship, political science, public policy, or law might be helpful in broadening the understanding of how to create change in the political realm. As evident at recent planetary health conferences, the community is already moving in this direction by inviting non-academic decision makers and speakers from such fields but most published work still concludes with a call to action rather than clear instructions as to how to take action.

Planetary health aims to encompass a global approach to health. However, the majority of contributions seem to come from wealthy, Anglophone countries (Figure 3), and most collaborations occur between a few wealthy countries (in this case, the US, UK, Australia, and Canada; see Supplementary Figure 1). Thus, steps are needed to increase global inclusion. Future efforts should focus on increasing scholarship from non-Western countries, such as hosting the 2021 Annual Meeting of the PHA in Brazil, as well as actively representing Indigenous knowledge. Encompassing different academic traditions and ways of knowing into the planetary health community has great potential to help the field grow and increase its impact. Indigenous knowledge is based in worldviews that are holistic and put emphasis on reciprocal relationships between humans and the planet—an understanding that is essential to sustainability concepts (33). As Redvers argues, “the problems of planetary health are both profound and complex; solutions can be found in a greater understanding of the self and the universe and the land as a medicine place” (15). There is currently a paucity in the planetary health field of work that considers Indigenous knowledge; only 7.4% (*n* = 20) of the articles and commentaries presented an in-depth consideration of Indigenous knowledge. By searching the peer-reviewed literature exclusively, we may have excluded Indigenous voices that are being expressed outside of an academic, Westernized publication system. To mitigate this issue at least partially, we intentionally included Indigenous databases in our literature search. However, since the inclusion of Indigenous voices is an explicit goal of the planetary health field, the paucity of publications that include Indigenous knowledge highlights room for improvement of inclusive publication strategies. Moreover, research done within Indigenous paradigms may not yet use the term planetary health because similar or identical ideas are already embedded in the respective worldview but are not made explicit as a specific field, which is a concept based on a Western idea of science.

### Strengths and Limitations

The strengths of this study include that it was conducted by an interdisciplinary, multi-lingual team, covering databases across multiple disciplines, including Indigenous databases. The content analysis was based on expert-driven themes, using a systematic approach to the literature search for the scoping review, and key results compared with those found in a bibliometric analysis. One limitation of our methodology is that—despite not restricting the language of publication—we only searched for the term planetary health in English, which could bias the publication countries and collaboration results. Another limitation is the subjective nature of content analysis. To mitigate this issue, several authors reviewed the same articles and reached a consensus through discussion when disagreement on the themes of a publication occurred. Finally, the bibliometric analysis of keyword co-occurrences was limited to a small sample size because the planetary health field is still relatively young and evolving. This means that the keyword network results may be sensitive to small changes in the sampling pool. Further studies using bibliometric analysis would be useful as the planetary health field continues to grow.

### Conclusion and Future Directions

The initiation of the Lancet Commission, the PHA, and other initiatives (e.g., *in VIVO*, London School of Hygiene and Tropical Medicine's Centre on Climate Change & Planetary Health) seems to positively affect the uptake of the concept of holistic health fields in general and planetary health in particular. Our study on the term planetary health in the peer-reviewed literature shows that funding and programs that encourage non-Anglophone and Indigenous knowledge representation within the field should be prioritized to increase breadth. Similarly, measures that encourage inter- and trans-disciplinary collaborations—particularly those incorporating non-health fields—may encourage authors of original research to use planetary health as a keyword and increase awareness of the field. Encouragingly, it appears that concepts not traditionally studied together, such as mental health and urban planning, are already being combined within planetary health. Since one of the goals of the field is to influence policy decisions, future research could focus on the impact that planetary health advocates may have on policy makers and strategies to do so effectively. Including the social sciences in general, and knowledge translation and political science in particular, into planetary health research provides opportunities for bridging the gap between academia and change makers. In the future, repeating this analysis over a longer time span will provide insight into the development of the field, the degree to which the term is being accepted in the academic environment, and the successes and failures of steps taken toward advancing the field of planetary health. Although it is important for the planetary health field to develop its own identity, its proponents should learn and form stronger coalitions with other thought leaders, including Indigenous ways of knowing, and other holistic health fields such as One Health and EcoHealth, to address many simultaneous threats, and to foster opportunities.

## Data Availability Statement

The original contributions presented in the study are included in the article/Supplementary Material, further inquiries can be directed to the corresponding author/s.

## Author Contributions

VR-R brought forward the initial idea and lead and contributed substantially to the emergence of the research question, the methodology, the literature search, the interpretation of results, and the preparation of manuscript and abstract. EA, FA-R, MC, SO, LS, and AH contributed equally and substantially to the development of the research question, the methodology, the literature search, and interpretation of results as well as the preparation of the manuscript and figures. FA-R, MC, and AH additionally led the efforts of the bibliometric analysis, conducted the analysis, and interpretation of the respective results as well as supported editing and revision of the manuscript. EA prepared the maps and figures included in this manuscript. All authors contributed to the article and approved the submitted version.

## Conflict of Interest

The authors declare that the research was conducted in the absence of any commercial or financial relationships that could be construed as a potential conflict of interest.

## Supplementary Material

Supplementary Figure 1 Map of country collaborations. This map shows one or more collaborations between country pairs.

Supplementary Table 1 List of all publications included in the final analysis of the scoping review on the use of the term “Planetary Health” in the peer-reviewed literature.
