# Supplementary figures and images for: Scoping Review and Bibliometric Analysis of the Term “Planetary Health” in the Peer-Reviewed Literature
Source: Front Public Health. 2020 Jul 29;8:343. doi: 10.3389/fpubh.2020.00343 (PMC7403469; doi:10.3389/fpubh.2020.00343)

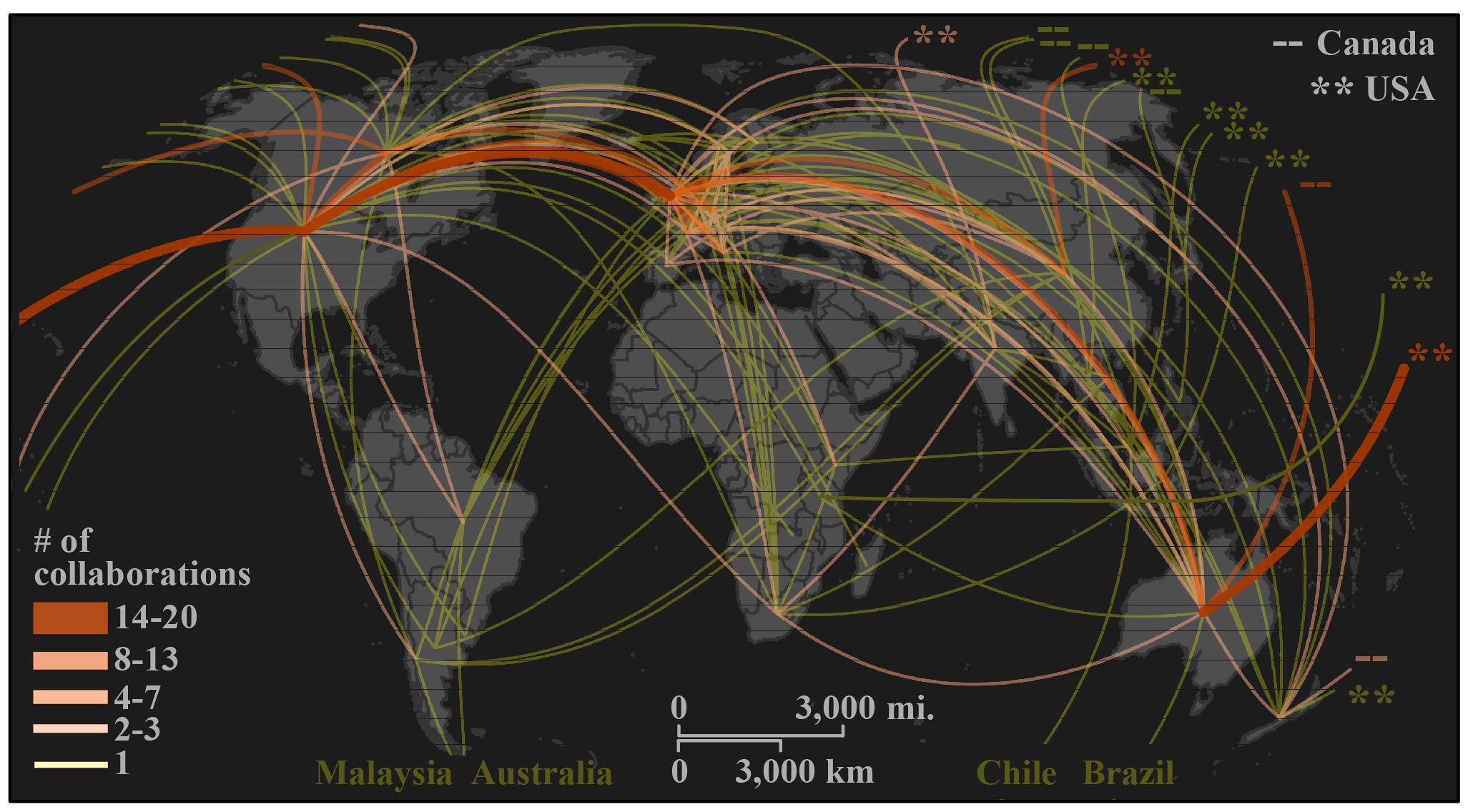

Supplement: Supplementary Figure 1 — Map of country collaborations. This map shows one or more collaborations between country pairs. [file Image_1.JPEG]
