# Supplementary material for: Scoping Review and Bibliometric Analysis of the Term “Planetary Health” in the Peer-Reviewed Literature
Source: Front Public Health. 2020 Jul 29;8:343. doi: 10.3389/fpubh.2020.00343 (PMC7403469; doi:10.3389/fpubh.2020.00343)
Supplement: Supplementary Table 1 — List of all publications included in the final analysis of the scoping review on the use of the term “Planetary Health” in the peer-reviewed literature. [file Table_1.pdf]

Supplementary Table 1. List of all publications included in the final analysis of the scoping review on the use of the term ‘Planetary Health’ in the peer-reviewed literature.

**Author(s)**	**Title**	**Year of publication**	**Journal/publisher**
Acunzo, David J; Escher, Gerard; Ottersen, Ole P; Whittington, Jason D; Gillet, Philippe; Stenseth, Nils C; Negrutiu, Ioan	Framing Planetary Health: arguing for resource-centred science	2018	The Lancet Planetary Health
Admasu, Kesetebirhan; Debessa, Kare	Action to protect human health from climate change: an African perspective	2015	The Lancet
Aginam, Obijiofor	Global governance approaches to Planetary Health in: One Planet, One Health	2019	Sydney University Press
Ağırbaşlı, Deniz; Ağırbaşlı, Mehmet	A Planetary Health lens for rare diseases in refugee populations and innovating systems medicine education	2019	OMICS: A Journal of Integrative Biology
Aguirre, Alonso; Basu, Niladri; Kahn, Laura H; Morin, Xenia K; Echaubard, Pierre; Wilcox, Bruce A; Beasley, Val R	Transdisciplinary and social-ecological health frameworks - Novel approaches to emerging parasitic and vector-borne diseases	2019	Parasite Epidemiology and Control
Al-Delaimy, Wael K	Planetary Health and population health: the Anthropocene requires different thinking and approaches in serving public health	2018	Current Environmental Health Reports
Alders, R; de Bruyn, J; Wingett, K; Wong, J	One Health, veterinarians and the nexus between disease and food security	2017	Australian Veterinary Journal
Alders, Robyn G	Achieving ethical and ecologically sustainable human diets through the Planetary Health paradigm	2017	Journal of Nutrition & Intermediary Metabolism
Alders, Robyn G; Ratanawongprasat, Natalie; Schönfeldt, Hettie; Stellmach, Darryl	A Planetary Health approach to secure, safe, sustainable food systems: workshop report	2018	Food Security
Almada, Amalia A; Golden, Christopher D; Osofsky, Steven A; Myers, Samuel S	A case for Planetary Health/GeoHealth	2017	Geoscience Data Journal
Almada, Amalia; Myers, Samuel S;	Planetary Health Alliance 2019 call for abstracts	2019	The Lancet Planetary Health

Golden, Christopher D; Burke, Katherine S; Luby, Stephen P			
Anonymous	Plenary 3-All for Health: the contribution of science to Planetary Health	2016	European Journal of Public Health
Anonymous	Power, politics and population health: the strength of collaboration	2018	MEDICC Review
Anonymous	Pushing for Planetary Health diets	2019	Food Australia
Anonymous	Vector control: time for a Planetary Health approach	2017	The Lancet Global Health
Asuzu, Michael C.; Onajole, Adebayo T.; Disu, Yahya	Public health at all levels in the recent Nigerian Ebola viral infection epidemic: lessons for community, public and international health action and policy	2015	Journal of Public Health Policy
Baker, Anthony	Report from ASM 2017: Planetary Health	2017	Microbiology Australia
Baldwin, Cathy; Rawstorne, Patrick	Public understanding of risk in health impact assessment: a psychosocial approach	2019	Impact Assessment and Project Appraisal
Barrett, M J; Alphonsus, Khrisha B; Harmin, Matt; Epp, Tasha; Hoessler, Carolyn; McIntyre, Danielle; Reeder, Bruce; Singh, Baljit	Learning for transdisciplinary leadership: why skilled scholars coming together is not enough	2019	BioScience
Beaglehole, Robert	Healthy planet, healthy people	2016	The Lancet
Beaglehole, Robert	Lethal but lawful: holding corporations to account	2014	The Lancet
Beauman, C.; Cannon, G.; Elmadfa, I.; Glasauer, P.; Hoffmann, I.; Keller, M.; Krawinkel, M.; Lang, T.; Leitzmann, C.; Lotsch, B.; Margetts, B. M.; McMichael, A. J.; Meyer-Abich, K.; Oltersdorf, U.; Pettoello-Mantovani, M.; Sabate, J.; Shetty, P.; Soria, M.; Spiekermann, U.; Tudge, C.; Vorster, H. H.; Wahlqvist, M.; Zerilli-Marimo, M.	The principles, definition and dimensions of the new nutrition science	2005	Public Health Nutrition

Behbod, Behrooz; Lauriola, Paolo; Leonardi, Giovanni; Crabbe, Helen; Close, Rebecca; Staatsen, Brigit; Knudsen, Lisbeth E.; de Hoogh, Kees; Medina, Sylvia; Semenza, Jan C.; Fletcher, Tony	Environmental and public health tracking to advance knowledge for Planetary Health	2016	European Journal of Public Health
Benatar, Solomon	Politics, power, poverty and global health: systems and frames	2016	International Journal of Health Policy & Management
Bernard, P.	Health psychology at the age of Anthropocene	2019	Health Psychology and Behavioral Medicine
Black, Daniel; Scally, Gabriel; Hunt, Alistair; Orme, Judy	We must look further upstream to enable Planetary Health-literate urban development	2018	The Lancet Planetary Health
Black, Daniel; Scally, Gabriel; Orme, Judy; Hunt, Alistair; Pilkington, Paul; Lawrence, Roderick; Ebi, Kristie	Moving health upstream in urban development: reflections on the operationalization of a transdisciplinary case study	2019	Global Challenges
Blackstone, Nicole Tichenor; El-Abbadi, Naglaa H; McCabe, Margaret S; Griffin, Timothy S; Nelson, Miriam E	Linking sustainability to the healthy eating patterns of the Dietary Guidelines for Americans: a modelling study	2018	The Lancet Planetary Health
Bosurgi, Raffaella; Horton, Richard	The Lancet Planetary Health: a new journal for a new discipline--a call for papers	2017	The Lancet
Boylan, S.; Syrett, K.; Colagiuri, R.	Role of law at the non-communicable diseases-climate change interface: considerations for planetary and population health policy	2013	Public Health
Braitstein, P.; Lama, T.; Keino, S.; Gladanac, B.; Yego, F.; Cole, D.; Tabu, J. S.; Cortinois, A.; Tarus, C.; Fox, A.	Increasing food security and nutrition resilience in response to climate change in east Africa: findings from a multisectoral symposium	2017	The Lancet Global Health
Brattig, Norbert W; Tanner, Marcel; Bergquist, Robert; Utzinger, Jurg	Impact of environmental changes on infectious diseases: key findings from an international conference in Trieste, Italy in May 2017	2019	Acta Tropica
Brousselle, Astrid; Butzbach, Camille	Redesigning public health for Planetary Health	2018	The Lancet Planetary Health

Brown, Alastair	Planetary Health digest	2019	The Lancet Planetary Health
Brown, Alastair; Hinsley, Sam	Planetary Health digest	2019	The Lancet Planetary Health
Brown, Andrew	Planetary Health	2013	Interdisciplinary Environmental Review
Bull, Fiona C; Hardman, Adrianne E	Walking: a best buy for public and Planetary Health	2018	British Journal of Sports Medicine
Buse, Chris G.; Oestreicher, Jordan Sky; Ellis, Neville R.; Patrick, Rebecca; Brisbois, Ben; Jenkins, Aaron P.; McKellar, Kaileah; Kingsley, Jonathan; Gislason, Maya; Galway, Lindsay; McFarlane, Ro A.; Walker, Joanne; Frumkin, Howard; Parkes, Margot	Public health guide to field developments linking ecosystems, environments and health in the Anthropocene	2018	Journal of Epidemiology and Community Health
Buse, Chris G; Smith, Maxwell; Silva, Diego S	Attending to scalar ethical issues in emerging approaches to environmental health research and practice	2019	Monash Bioethics Review
Butler, Colin D	Limits to growth, planetary boundaries, and Planetary Health	2017	Current Opinion in Environmental Sustainability
Butler, Colin D	Philanthrocapitalism: promoting global health but failing Planetary Health	2019	Challenges
Butler, Colin D	Planetary epidemiology: towards first principles	2018	Current Environmental Health Reports
Campostrini, Stefano; Guerra, Ranieri	Can the scientific world positively influence decision makers on Planetary Health?	2018	The Lancet
Canavan, Chelsey R.; Noor, Ramadhani A.; Golden, Christopher D.; Juma, Calestous; Fawzi, Wafaie	Sustainable food systems for optimal Planetary Health	2017	Transactions of the Royal Society of Tropical Medicine and Hygiene
Cannon, Geoffrey; Leitzmann, Claus	The new nutrition science project	2005	Public Health Nutrition
Caoili, Salvador Eugenio	Antibodies, synthetic peptides and related constructs for Planetary Health based on green chemistry in the Anthropocene	2018	Future science OA
Cape, John; Mack, Jessica; Gomez, Jacob	A new leadership approach for Planetary Health	2017	The Lancet Planetary Health

Capon, Anthony	Harnessing urbanisation for human wellbeing and Planetary Health	2017	The Lancet Planetary Health
Capon, Anthony G.; AC Talley, Nicholas J.; Horton, Richard C.;	Planetary Health: what is it and what should doctors do?	2018	Medical Journal of Australia
Carlson, Colin J; Trisos, Christopher H	Climate engineering needs a clean bill of health	2018	Nature Climate Change
Chahbazi, John; Grow, Shelly	Common foods and farming methods thought to promote health: what the data show	2008	Primary Care
Chen, Chong; Nakagawa, Shin	Planetary Health and the future of human capacity: the increasing impact of planetary distress on the human brain	2018	Challenges
Clark, Helen	Governance for Planetary Health and sustainable development	2015	The Lancet
Cole, Jennifer; Bickersteth, Sam	What's planetary about health? An analysis of topics covered in The Lancet Planetary Health's first year	2018	The Lancet Planetary Health
Costa, Rosa; Alders, Robyn G.; Rukambile, Elpidius; Magoke, Godfrey; Dumas, Sarah E.; Maulaga, Wende; Jong, Joanita	Family poultry: Multiple roles, systems, challenges, and options for sustainable contributions to household nutrition security through a Planetary Health lens	2018	Maternal & Child Nutrition
Davidson, Patricia M.	Rockefeller Foundation-Lancet Commission report: A call to action for human health	2016	International Journal of Nursing Studies
De Paula, Nicole	What is Planetary Health? Addressing the environment-health nexus in Southeast Asia in the era of the Sustainable Development Goals: opportunities for international relations scholars	2018	Revista Brasileira De Politica Internacional
de Sa, Thiago Hérick	Can Coca Cola promote physical activity?	2014	The Lancet
Delany, Louise; Signal, Louise; Thomson, George	International trade and investment law: a new framework for public health and the common good	2018	BMC Public Health
Demaio, Alessandro R.; Rockström, Johan	Human and Planetary Health: towards a common language	2015	The Lancet
Duane, Brett; Harford, Sara; Ramasubbu, Darshini; Stancliffe, Rachel; Pasdeki-Clewer, Eleni; Lomax, Richard; Steinbach, Inge	Environmentally sustainable dentistry: a brief introduction to sustainable concepts within the dental practice	2019	British Dental Journal

Duboz, Raphael; Echaubard, Pierre; Promburom, Panomsak; Kilvington, Margaret; Ross, Helen; Allen, Will; Ward, John; Deffuant, Guillaume; de Garine-Wichatitsky, Michel; Binot, Aurelie	Systems thinking in practice: participatory modeling as a foundation for integrated approaches to health	2018	Frontiers in Veterinary Science
Dune, Tinashe; Thomas Astell-Burt; Rubab Firdaus	The built environment and sexual and reproductive health	2017	Australian and New Zealand Journal of Public Health
Dusheck, Jennie	Worried about climate change? Ask your doctor	2019	Frontiers in Ecology and the Environment
Ebi, Kristie L.; Frumkin, Howard; Hess, Jeremy J.	Protecting and promoting population health in the context of climate and other global environmental changes	2017	Anthropocene
Emerich, Monica	The spirituality of sustainability: healing the self to heal the world through healthy living media	2006	[Doctoral thesis]
Eshel, Gidon; Martin, Pamela	Alignment of personal and Planetary Health: beyond greenhouse gases alone	2009	Epidemiology
Ezeh, Alex	Addressing Planetary Health challenges in Africa	2016	Public Health Reviews
Fernandez Ortiz, Anna; Bueno Ortiz, Jose Miguel	[Commitment to Planetary Health]	2019	Atención Primaria
Fernando, Julian W.; O'Brien, Léan V.; Judge, Madeline; Kashima, Yoshihisa	More than idyll speculation: Utopian thinking for Planetary Health	2019	Challenges
Filippelli, Gabriel M	Exploring the paradox of increased global health and degraded global environment: how much borrowed time is humanity living on?	2018	Geoscience Data Journal
Fisher, Jack E; Pedersen, Marie; Loft, Steffen; Andersen, Zorana J	Opportunities and challenges within urban health and sustainable development	2017	Current Opinion in Environmental Sustainability
Flowra, Mahfuza Talukder; Asaduzzaman, Muhammad	Resurgence of infectious diseases due to forced migration: is Planetary Health and One Health action synergistic?	2018	The Lancet Planetary Health
Fontana, Luigi; Atella, Vincenzo; Kammen, Daniel M	Energy efficiency as a unifying principle for human, environmental, and global health	2013	F1000 Research

Foster, Alexander; Cole, Jennifer; Farlow, Andrew; Petrikova, Ivica	Planetary Health ethics: beyond first principles	2019	Challenges
Frank-White, Nathaniel Etheridge; Burns-Kirkness, Rowan; Frank, Erica	The effect of dietary and transportation choices on climate change	2011	Food and Nutrition Sciences
Friel, S; Bowen, K; Campbell-Lendrum, D; Frumkin, H; McMichael, A J; Rasanathan, K	Climate change, noncommunicable diseases, and development: the relationships and common policy opportunities	2011	Annual Review of Public Health
Frumkin, Howard; Haines, Andy	Global environmental change and noncommunicable disease risks	2019	Annual Review of Public Health
Frumkin, Howard; Watts, Nicholas	Health, science, faith, and stewardship	2018	EcoHealth
Fugelli, Per	Death and Planetary Health	2016	The Lancet
Gabrysch, Sabine	Imagination challenges in Planetary Health: re-conceptualising the human-environment relationship	2018	The Lancet Planetary Health
Gagliano, Monica	Planetary Health: are we part of the problem or part of the solution?	2018	Challenges
Gagné, Thierry; Lapalme, Josée	Multidisciplinarity in health promotion and the era of Planetary Health	2019	Health Promotion International
Garchitorena, A; Sokolow, SH; Roche, B; Ngonghala, C N; Jocque, M; Lund, A; Barry, M; Mordecai, E A; Daily, G C; Jones, J H; Andrews, J R; Bendavid, E; Luby, S P; LaBeaud, AD; Seetah, K; Guegan, J F; Bonds, M H; De Leo, GA	Disease ecology, health and the environment: a framework to account for ecological and socio-economic drivers in the control of neglected tropical diseases	2017	Philosophical Transactions of the Royal Society of London, Series B: Biological Sciences
Garg, Ashish; Mithal, Varun; Chamber, Yashu; Brugere, Ivan; Chaudhari, Vijay; Dunham, Marc; Krishna, Vikrant; Krishnamurthy, Sairam; Vangala, Sruthi; Boriah, Shyam; Steinbach, Michael; Kumar, Vipin; Cho, Albert; Stanley, J.	GOPHER: global observation of Planetary Health and ecosystem resources	2011	2011 Ieee International Geoscience and Remote Sensing Symposium

D.; Abraham, Teji; Castilla-Rubio, Juan Carlos; Potter, Christopher; Klooster, Steven			
Geiger, Sonja M.; Otto, Siegmar; Schrader, Ulf	Mindfully green and healthy: an indirect path from mindfulness to ecological behavior	2018	Frontiers in Psychology
Giles-Corti, Billie; Lowe, Melanie; Arundel, Jonathan	Achieving the SDGs: evaluating indicators to be used to benchmark and monitor progress towards creating healthy and sustainable cities	2019	Health Policy
Gill, Stephen R.; Benatar, Solomon R.	Reflections on the political economy of Planetary Health	2019	Review of International Political Economy
Goldberg, Tony L.; Patz, Jonathan A.	The need for a global health ethic	2015	The Lancet
Golden, Christopher D.; Seto, Katherine L.; Dey, Madan M.; Chen, Oai L.; Gephart, Jessica A.; Myers, Samuel S.; Smith, Matthew; Vaitla, Bapu; Allison, Edward H.	Does aquaculture support the needs of nutritionally vulnerable nations?	2017	Frontiers in Marine Science
Golden, Christopher D; Vaitla, Bapu; Ravaoliny, Laurent; Vonona, Miadana A; EJ Gasta Anjaranirina; Randriamady, Hervet J; Glahn, Raymond P; Guth, Sarah E; Fernald, Lia CH; Myers, Samuel S	Seasonal trends of nutrient intake in rainforest communities of north-eastern Madagascar	2019	Public Health Nutrition
Goodman, B	The debate on climate change and health in the context of ecological public health: a necessary corrective to Costello et al's 'biggest global health threat', or co-opted apologists for the neoliberal hegemony?	2014	Public Health
Goodman, Benny	Developing the concept of sustainability in Nursing	2016	Nursing Philosophy
Gustafson, Craig	Tieraona Low Dog, MD: the many aspects of greening medicine	2015	Alternative Therapies in Health & Medicine
Haahtela, Tari	A biodiversity hypothesis	2019	Allergy
Hadjikakou, Michalis	Trimming the excess: environmental impacts of	2017	Ecological Economics

	discretionary food consumption in Australia		
Haines, Andy	Addressing challenges to human health in the Anthropocene epoch—an overview of the findings of the Rockefeller/Lancet Commission on Planetary Health	2016	Public Health Reviews
Haines, Andy; Hanson, Craig; Ranganathan, Janet	Planetary Health Watch: integrated monitoring in the Anthropocene epoch	2018	The Lancet Planetary Health
Haines, Andy; Whitmee, Sarah; Horton, Richard	Planetary Health: a call for papers	2014	The Lancet
Hancock, Trevor	Beyond science and technology: creating Planetary Health needs not just 'head stuff', but social engagement and 'heart, gut and spirit' stuff	2019	Challenges
Hancock, Trevor; Capon, Anthony; Dooris, Mark; Patrick, Rebecca	One planet regions: Planetary Health at the local level	2017	The Lancet Planetary Health
Hays, Richard	Including health promotion and illness prevention in medical education: a progress report	2018	Medical Education
Hernando-Amado, Sara; Coque, Teresa M; Baquero, Fernando; Martinez, Jose L	Defining and combating antibiotic resistance from One Health and global health perspectives	2019	Nature Microbiology
Hill-Cawthorne, Grant A.	One Health/EcoHealth/Planetary Health and their evolution in: One Planet, One Health	2019	Sydney University Press
Hill-Cawthorne, Grant A; Capon, Anthony G; Sorrell, Tania C; Marais, Ben J	A Planetary Health approach to emerging infections in Australia	2017	The Lancet
Hill-Cawthorne, Grant; Negin, Joel; Capon, Tony; Gilbert, Gwendolyn L; Nind, Lee; Nunn, Michael; Ridgway, Patricia; Schipp, Mark; Firman, Jenny; Sorrell, Tania C; Marais, Ben J	Advancing Planetary Health in Australia: focus on emerging infections and antimicrobial resistance	2019	BMJ Global Health
Hinsley, Sam	Planetary Health digest	2019	The Lancet Planetary Health
Horton, Richard	Offline: Australia leads on climate and health	2015	The Lancet

Horton, Richard	Offline: Medicine's theory of relativity	2015	The Lancet
Horton, Richard	Offline: Planetary Health--a new vision for the post-2015 era	2013	The Lancet
Horton, Richard	Offline: Planetary Health--gains and challenges	2016	The Lancet
Horton, Richard	Offline: Planetary Health's next frontier-biodiversity	2017	The Lancet
Horton, Richard	Offline: Planetary Health--the great acceleration	2017	The Lancet
Horton, Richard	Offline: Planetary Health—where next?	2016	The Lancet
Horton, Richard	Offline: Planetary Health—worth everything	2018	The Lancet
Horton, Richard	Offline: Progress towards Planetary Health	2015	The Lancet
Horton, Richard	Offline: Reimagining the meaning of health	2014	The Lancet
Horton, Richard	Offline: The health of our societies is in peril	2018	The Lancet
Horton, Richard	Offline: Why the unity of life matters for our Planetary Health	2015	The Lancet
Horton, Richard; Beaglehole, Robert; Bonita, Ruth; Raeburn, John; McKee, Martin; Wall, Stig	From public to Planetary Health: a manifesto	2014	The Lancet
Horton, Richard; Beaglehole, Robert; Bonita, Ruth; Raeburn, John; McKee, Martin; Wall, Stig	The Federation's pages - Planetary Health and the World Federation of Public Health Associations	2014	Journal of Public Health Policy
Horton, Richard; Lo, Selina	Planetary Health: a new science for exceptional action	2015	The Lancet
Horwitz, Pierre; Parkes, Margot W.	Intertwined strands for ecology in Planetary Health	2019	Challenges
Houlton, Benjamin Z; Almaraz, Maya; Aneja, Viney; Austin, Amy T; Bai, Edith; Cassman, Kenneth G; Compton, Jana E; Davidson, Eric A; Erisman, Jan Willem; Galloway, James N; Gu, Baojing; Yao, Guolin; Martinelli, Luiz A; Scow, Kate; Schlesinger, William H; Tomich, Thomas P;	A world of cobenefits: solving the global nitrogen challenge	2019	Earth's Future

Wang, Chao; Zhang, Xin			
Hudson-Edwards, Karen A.	Mining and Planetary Health: a GeoHealth - led special collection	2018	Geoscience Data Journal
Iacobucci, Gareth	Planetary Health: WONCA urges family doctors to commit to action	2019	BMJ (Clinical research ed.)
India State-Level Disease Burden Initiative Air Pollution Collaborators	The impact of air pollution on deaths, disease burden, and life expectancy across the states of India: the Global Burden of Disease Study 2017	2019	The Lancet Planetary Health
Jenkins, Aaron; Capon, Anthony; Negin, Joel; Marais, Ben; Sorrell, Tania; Parkes, Margot; Horwitz, Pierre	Watersheds in Planetary Health research and action	2018	The Lancet Planetary Health
Johnson, Hope	Eating for health and the environment: Australian regulatory responses for dietary change	2015	QUT Law Review
Johnston, Nancy; Rogers, Martha; Cross, Nadine; Sochan, Anne	Global and Planetary Health: teaching as if the future matters	2005	Nursing Education Perspectives
Kadandale, Sowmya; Marten, Robert; Smith, Richard	The palm oil industry and noncommunicable diseases	2019	Bulletin of the World Health Organization
Kahn, Laura H; Kaplan, Bruce; Monath, Thomas; Woodall, Jack; Conti, Lisa	A manifesto for Planetary Health	2014	The Lancet
Kamp, David	Letter to the Editors	2016	Health Environments Research & Design Journal
Katz, David L; Doughty, Kimberly N; Geagan, Kate; Jenkins, David A; Gardner, Christopher D	Perspective: The public health case for modernizing the definition of protein quality	2019	Advances in Nutrition
Kilic, Hakan	Migration studies: a 21st century scholarship shaping human and Planetary Health	2019	OMICS: A Journal of Integrative Biology
Kingsley, Jonathan; O'Brien, Jacqueline; Bailey, Aisling	Coffee consumers in university settings and Planetary Health	2018	The Lancet Planetary Health
Kirby, Tony	Tony Capon: the world's first professor of Planetary Health	2018	The Lancet
Koger, Susan M.; Scott, Britain A.	Teaching environmentally focused psychology	2012	Ecopsychology

Kraak, Vivica I.1, vivica51@vt.edu	Skills and tools for public health professionals to address corporate power to protect human and Planetary Health	2018	American Journal of Public Health
Kunz, Thomas H; Gauthreaux, Sidney A; Hristov, Nickolay I; Horn, Jason W; Jones, Gareth; Kalko, Elisabeth K V; Larkin, Ronald P; McCracken, Gary F; Swartz, Sharon M; Srygley, Robert B; Dudley, Robert; Westbrook, John K; Wikelski, Martin	Aeroecology: probing and modeling the aerosphere	2008	Integrative and Comparative Biology
Kurth, Ann E.	Planetary Health and the role of Nursing: a call to action	2017	Journal of Nursing Scholarship
Landires, Ivan; Nuñez-Samudio, Virginia; Apraez-Ippolito, Giovanni; Castro, Guillermo	Planetary Health: a new sociopolitical framework is urgently needed	2018	The Lancet
Lang, Tim	Reshaping the food system for ecological public health	2009	Journal of Hunger & Environmental Nutrition
Leffers, Jeanne; Levy, Ruth McDermott; Nicholas, Patrice K.; Sweeney, Casey F.	Mandate for the Nursing profession to address climate change through Nursing education	2017	Journal of Nursing Scholarship
Lehmkuhl, Dieter	Das Thema Klimawandel und seine Bedeutung im Gesundheitssektor: Entwicklung, Akteure, Meilensteine	2019	Bundesgesundheitsblatt - Gesundheitsforschung - Gesundheitsschutz
Lerner, Henrik; Berg, Charlotte	A comparison of three holistic approaches to health: One Health, EcoHealth, and Planetary Health	2017	Frontiers in Veterinary Science
Liutsko, Liudmila	The integrative model of personality and the role of personality in a Planetary Health context	2019	Personality and Individual Differences
Lo, Selina N.; Ferguson, Kaiya; Kayak, Eugenie; Faulkner, Kingsley	Planetary Health: the Australian chapter	2018	Medical Journal of Australia
Logan, Alan C.; Goldenberg, Joshua Z.; Guiltinan, Jane; Seely, Dugald; Katz, David L.	North American naturopathic medicine in the 21st century: time for a seventh guiding principle - Scientia Critica	2018	Explore: The Journal of Science & Healing
Logan, Alan C; Prescott, Susan L	Astrofood, priorities and pandemics: reflections of an ultra-	2017	Challenges

	processed breakfast program and contemporary dysbiotic drift		
Logan, Alan C; Prescott, Susan L; Haahtela, Tari; Katz, David L	The importance of the exposome and allostatic load in the Planetary Health paradigm	2018	Journal of Physiological Anthropology
Macaux, William P.	Generative leadership: responding to the call for responsibility	2012	Journal of Management Development
Maini, Rishma; Law, Ronald; Duque, Francisco 3rd; Balboa, Gloria; Noda, Hiroyuki; Nakamura, Sachiko; Murray, Virginia	Monitoring progress towards Planetary Health	2017	BMJ (Clinical research ed.)
Marsden, Dawn	The cure is in the cause: a rationale and guide for scaling up Indigenous principles and practices for resilience and sustainability	2017	Journal of Sustainability Education
Martin, Debbie; Ratima, Mihi, mihiratima@gmail.com; Delormier, Treena; Castleden, Heather	Indigenous voices and knowledge systems – promoting Planetary Health, health equity, and sustainable development now and for future generations	2019	Global Health Promotion
Mash, Robert	The Anthropocene - the biggest threat to health on the African continent	2019	African Journal of Primary Health Care & Family Medicine
Meltzer, Helle Margrete; Brantsæter, Anne Lise; Trolle, Ellen; Eneroth, Hanna; Fogelholm, Mikael; Ydersbond, Trond Arild; Birgisdottir, Bryndis Eva	Environmental sustainability perspectives of the Nordic diet	2019	Nutrients
Middleton, John	Public health in England in 2016 - the health of the public and the public health system: a review	2017	British Medical Bulletin
Miller, Elise; Valenti, Maria	Healthy environments across generations = healthy aging	2014	International Journal of Aging & Human Development
Minos, Dimitrios; Butzlaff, Iris; Demmler, Kathrin Maria; Rischke, Ramona	Economic growth, climate change, and obesity	2016	Current Obesity Reports
Mishra, Shiva Raj; Adhikari, Bipin	Planetary Health in Nepal's post-earthquake rebuilding agenda: progress and future directions	2019	The Lancet Planetary Health
Molton, Sarah	The role of funders: Wellcome Trust	2016	Public Health Reviews

Moore, Michael; McKee, Martin; Borisch, Bettina; Ricciardi, Walter	The global charter for the public's health	2016	European Journal of Public Health
Moran, Dominic	Antimicrobial use and Planetary Health: developing a framework for priorities	2018	The Lancet Planetary Health
Morand, Serge	Anthropogenic global defaunation and its consequences for Planetary Health	2018	International Journal of Infectious Diseases
Morand, Serge; Lajaunie, Claire	Linking biodiversity with health and well-being: consequences of scientific pluralism for ethics, values and responsibilities	2019	Asian Bioethics Review
Morisetti, Neil; Blackstock, Jason J.	Impact of a changing climate on global stability, wellbeing, and Planetary Health	2017	The Lancet Planetary Health
Moser, Adrian Mathias; Stigler, Florian L; Haditsch, Bernd	Physicians' responsibility for Planetary Health	2017	The Lancet Planetary Health
Moysés, Samuel J; Soares, Renata C	Planetary Health in the Anthropocene	2019	Health Promotion International
Myers, Samuel S	Planetary Health: protecting human health on a rapidly changing planet	2017	The Lancet
Myers, Samuel S.; Smith, Matthew R.; Guth, Sarah; Golden, Christopher D.; Vaitla, Bapu; Mueller, Nathaniel D.; Dangour, Alan D.; Huybers, Peter	Climate change and global food systems: potential impacts on food security and undernutrition	2017	Annual Review of Public Health
Myers, Samuel S; Golden, Christopher D; Almada, Amalia A; Grant, Liz; Planetary Health Alliance	Planetary Health research in 2018: a call for abstracts	2017	The Lancet Planetary Health
Myers, Samuel S; Golden, Christopher D; Almada, Amalia A; Osofsky, Steven A; Pongsiri, Montira J	Research in Planetary Health: a call for abstracts	2016	The Lancet
Nair, Amruta; Priya, S.	Andrew Paul Haines	2019	Current Science
Needoba, Joseph A; Tratnyek, Paul G	Planetary Health thematic web collection	2018	Environmental Science - Processes & Impacts
Nelson, David H.; Prescott, Susan L.; Logan, Alan C.; Bland, Jeffrey S.	Clinical Ecology - transforming 21st-century medicine with Planetary Health in mind	2019	Challenges

Nicholas, Patrice K; Breakey, Suellen	The economics of climate change and the intersection with conflict, violence, and migration: implications for the Nursing profession	2019	Nursing Economics
Ooms, Gorik; Pas, Remco van de; Decoster, Kristof; Hammonds, Rachel	Thinking out of the box: a green and social climate fund comment on "Politics, power, poverty and global health: systems and frames"	2016	International Journal of Health Policy and Management
Osofsky, Steven A; Pongsiri, Montira J	Operationalising Planetary Health as a game-changing paradigm: health impact assessments are key	2018	The Lancet Planetary Health
Oxman, Andrew D	Key concepts for making informed choices	2019	Nature
Parkes, Margot W.; Allison, Sandra; Harder, Henry G.; Hoogeveen, Dawn; Kutzner, Diana; Aalhus, Melissa; Adams, Evan; Beck, Lindsay; Brisbois, Ben; Buse, Chris G.; Chiasson, Annika; Cole, Donald C.; Dolan, Shayna; Fauré, Anne; Fumerton, Raina; Gislason, Maya K.; Hadley, Louisa; Hallström, Lars K.; Horwitz, Pierre; Marks, Raissa	Addressing the environmental, community, and health impacts of resource development: challenges across scales, sectors, and sites	2019	Challenges
Pascal, Mathilde; Beaudeau, Pascal; Medina, Sylvia; Hamilton, Nikita Charles	Global change: a public health researcher’s ethical responsibility	2019	Current Environmental Health Reports
Passarelli, Simone; Canavan, Chelsey R.; Paarlberg, Robert; Juma, Calestous; Akyeampong, Emmanuel; Fekadu, Habtamu; Golden, Christopher D.; Gunaratna, Nilupa S.; Jaacks, Lindsay M.; Kennedy, Eileen; Madzorera, Isabel; McCann, James;	Harnessing university strengths in multisectoral collaborations for Planetary Health	2018	Current Developments in Nutrition

Nwanze, Kanayo; Sauerborn, Rainer; Tang, Lixia; Webb, Patrick; Willett, Walter C.; Fawzi, Wafaie W.			
Patrick, Rebecca; Armstrong, Fiona; Hancock, Trevor; Capon, Anthony; Smith, James A.	Climate change and health promotion in Australia: navigating political, policy, advocacy and research challenges	2019	Health Promotion Journal of Australia
Pattanayak, S. K.; Kramer, R. A.; Vincent, J. R.	Ecosystem change and human health: implementation economics and policy	2017	Philosophical Transactions of the Royal Society B: Biological Sciences
Pattanayak, Subhrendu K; Haines, Andy	Implementation of policies to protect Planetary Health	2017	The Lancet Planetary Health
Perez-Escamilla, Rafael	Food insecurity and the erosion of human and Planetary Health: is there light at the end of the tunnel?	2018	American Journal of Human Biology
Perez-Escamilla, Rafael	Food security and the 2015-2030 Sustainable Development Goals: from human to Planetary Health: perspectives and opinions	2017	Current Developments in Nutrition
Pongsiri, Montira J; Bickersteth, Sam; Colon, Cristina; DeFries, Ruth; Dhaliwal, Mandeep; Georgeson, Lucien; Haines, Andrew; Linou, Natalia; Murray, Virginia; Naeem, Shahid; Small, Roy; Ungvari, Judit	Planetary Health: from concept to decisive action	2019	The Lancet Planetary Health
Pongsiri, Montira J; Gatzweiler, Franz W; Bassi, Andrea M; Haines, Andy; Demassieux, Fanny	The need for a systems approach to Planetary Health	2017	The Lancet Planetary Health
Poppy, Guy M; Baverstock, Jenny	Rethinking the food system for human health in the Anthropocene	2019	Current Biology
Potter, John D	Monocultures - a blight on human and Planetary Health in: Health	2015	ANU Press
Potter, John D	Red and processed meat, and human and Planetary Health of People, Places and Planet	2017	BMJ (Clinical research ed.)
Potter, Teddie	Planetary Health: the next frontier in Nursing education	2019	Creative Nursing

Pradyumna, Adithya	Planetary Health and food systems: insights from global SDGs	2018	The Lancet Planetary Health
Prescott, Susan L.; Kozyrskyj, Anita L.; Logan, Alan C.; Campbell, Dianne E.; Albrecht, Glenn; Kollman, Tobias R.; Takaro, Tim K.; Bosco, Anthony; Silva, Desiree; Lowry, Christopher A.; Olsen, Jonathan R.; Ellaway, Anne; McCrorie, Paul; Mitchell, Richard; Capaldi, Colin A.; Robinson, Jake M.; Daisley, Brendan A.; Trinder, Mark; McDowell, Tim W.; Welle, Hylke	Seventh annual conference of inVIVO Planetary Health on transforming life: unify personal, public, and Planetary Health	2018	Challenges
Prescott, Susan L.; Logan, Alan C.	Narrative medicine meets Planetary Health: mindsets matter in the Anthropocene	2019	Challenges
Prescott, Susan L.; Logan, Alan C.	Planetary Health: from the wellspring of holistic medicine to personal and public health imperative	2019	Explore: The Journal of Science & Healing
Prescott, Susan L.; Logan, Alan C.; Albrecht, Glenn; Campbell, Dianne E.; Crane, Julian; Cunsolo, Ashlee; Holloway, John W.; Kozyrskyj, Anita L.; Lowry, Christopher A.; Penders, John; Redvers, Nicole; Renz, Harald; Stokholm, Jakob; Svanes, Cecilie; Wegienka, Ganesa	The Canmore Declaration: statement of principles for Planetary Health	2018	Challenges
Prescott, Susan L.; Logan, Alan C.; Katz, David L.	Preventive medicine for person, place, and planet: revisiting the concept of high-level wellness in the Planetary Health paradigm	2019	International Journal of Environmental Research and Public Health
Prescott, Susan L.; Wegienka, Ganesa; Logan, Alan C.; Katz, David L.	Dysbiotic drift and biopsychosocial medicine: how the microbiome links personal, public and Planetary Health	2018	BioPsychoSocial Medicine

Prescott, Susan L; Logan, Alan C	Down to Earth: Planetary Health and biophilosophy in the Symbiocene epoch	2017	Challenges
Prescott, Susan L; Logan, Alan C	Larger than life: injecting hope into the planetary eealth paradigm	2018	Challenges
Prescott, Susan L; Pearson, Glenn; Snook, Jamie; Cunsolo, Ashlee	On hume and Planetary Health	2018	The Lancet
Prior, Jason H; Connon, Irena Lc; McIntyre, Erica; Adams, Jon; Capon, Anthony; Kent, Jennifer; Rissel, Chris; Thomas, Leena E; Thompson, Susan M; Westcott, Harriet	Built environment interventions for human and Planetary Health: integrating health in climate change adaptation and mitigation	2018	Public Health Research & Practice
Qiao, Min; Ying, Guang-Guo; Singer, Andrew C.; Zhu, Yong-Guan	Review of antibiotic resistance in China and its environment	2018	Environment International
Rabinowitz, Peter MacGarr; Pappaioanou, Marguerite; Bardosh, Kevin Louis; Conti, Lisa	A planetary vision for one health	2018	BMJ Global Health
Ratima, Mihi; Martin, Debbie; Castleden, Heather; Delormier, Treena	Indigenous voices and knowledge systems - promoting Planetary Health, health equity, and sustainable development now and for future generations	2019	Global Health Promotion
Rayner, Geof; Gracia, Mabel; Young, Elizabeth; Mauleon, Jose R; Luque, Emilio; Rivera-Ferre, Marta G	Why are we fat? Discussions on the socioeconomic dimensions and responses to obesity	2010	Globalization and Health
Reddy, K Srikanth; Roberts, Janet Hatcher	Mitigating air pollution: Planetary Health awaits a cosmopolitan moment	2019	The Lancet Planetary Health
Redvers, Nicole	The value of global Indigenous knowledge in Planetary Health	2018	Challenges
Reynolds, J. P.; Archer, S.; Pilling, M.; Kenny, M.; Hollands, G. J.; Marteau, T. M.	Public acceptability of nudging and taxing to reduce consumption of alcohol, tobacco, and food: a population-based survey experiment	2019	Social Science & Medicine
Rideout, Karen; Kosatsky, Tom	Fish for dinner? Balancing risks, benefits, and values in formulating food consumption advice	2017	Risk Analysis

Robinson, Jake M.; Breed, Martin F.	Green prescriptions and their co-benefits: integrative strategies for public and environmental health	2019	Challenges
Robinson, Jake M.; Mills, Jacob G.; Breed, Martin F.	Walking ecosystems in microbiome-inspired green infrastructure: an ecological perspective on enhancing personal and Planetary Health	2018	Challenges
Rockefeller Jr., David	The role of foundations: Rockefeller Foundation	2016	Public Health Reviews
Rosa, William E.; Upvall, Michele J.	The case for a paradigm shift: from global to planetary Nursing	2019	Nursing Forum
Rosa, William E; Dossey, Barbara M; Watson, Jean; Beck, Deva-Marie; Upvall, Michele J	The United Nations Sustainable Development Goals: the ethic and ethos of holistic Nursing	2019	Journal of Holistic Nursing: Official Journal of the American Holistic Nurses' Association
Rosa, William E; Kurth, Ann E; Sullivan-Marx, Eileen; Shamian, Judith; Shaw, Holly K; Wilson, Lynda L; Crisp, Nigel	Nursing and midwifery advocacy to lead the United Nations Sustainable Development Agenda	2019	Nursing Outlook
Rose, Diego; Willits-Smith, Amelia; Heller, Martin	Diet and Planetary Health: single-item substitutions significantly reduce the carbon footprint of self-selected diets reported in NHANES	2019	Current Developments in Nutrition
Rosencrans, Kendra	Nurturing faith for action: theological education and global responsibility	2014	Dialog: A Journal of Theology
Ruff, Tilman A.	WFPHA: World federation of public health associations	2018	Journal of Public Health Policy
Ryan, Jacinta T; Jones, Sandra; Hayes, Peter A; Craig, Jeffrey M	Leadership evolution for Planetary Health: a genomics perspective	2019	Challenges
Salk, Jonathan D.	Planetary Health: a new reality	2019	Challenges
Sanchez-Sabate, Ruben; Sabate, Joan	Consumer attitudes towards environmental concerns of meat consumption: a systematic review	2019	International Journal of Environmental Research and Public Health
Saunders, Patrick J.; Middleton, John D.	Response to Behbod B et al Environmental and public health tracking to advance knowledge for Planetary Health	2017	European Journal of Public Health
Scatigna, M; D'Eugenio, S; Cesarini, V; Coppola, L; Lemma, P; Fabiani, L; Romano Spica, V; WDPP,	Physical activity as a key issue for promoting human health on a local and global scale: evidences and perspectives	2019	Annali di Igiene: Medicina Preventiva e di Comunita

Working Group Doping Prevention Project			
Schenk, Elizabeth C.	Environmental stewardship in Nursing: introducing the "WE ACT– PLEASE" framework	2019	Creative Nursing
Schuftan, Claudio; Legge, David; Sanders, David; Nadimpally, Sarojini	A manifesto for Planetary Health	2014	The Lancet
Schultz, Rosalie	Implementation of policies to protect Planetary Health	2018	The Lancet Planetary Health
Schutte, Stefanie; Gemenne, Francois; Zaman, Muhammad; Flahault, Antoine; Depoux, Anneliese	Connecting Planetary Health, climate change, and migration	2018	The Lancet Planetary Health
Schwartz, Brian S; Parker, Cindy; Glass, Thomas A; Hu, Howard	Global environmental change: what can health care providers and the environmental health community do about it now?	2006	Environmental Health Perspectives
Seltenrich, Nate	Down to Earth: the emerging field of Planetary Health	2018	Environmental Health Perspectives (Online)
Shrivastava, Paul; Raivio, Kari; Kasuga, Fumiko; Tewksbury, Joshua; Haines, Andy; Daszak, Peter	Future Earth Health Knowledge-Action Network	2016	Public Health Reviews
Singer, Merrill	The spread of Zika and the potential for global arbovirus syndemics	2017	Global Public Health
Siri, Jose Gabriel	Sustainable, healthy cities: making the most of the urban transition	2016	Public Health Reviews
Sokolow, Susanne H; Jones, Isabel J; Jocque, Merlijn; La, Diana; Cords, Olivia; Knight, Anika; Lund, Andrea; Wood, Chelsea L; Lafferty, Kevin D; Hoover, Christopher M; Collender, Phillip A; Remais, Justin V; Lopez-Carr, David; Fisk, Jonathan; Kuris, Armand M; De Leo, Giulio A	Nearly 400 million people are at higher risk of schistosomiasis because dams block the migration of snail-eating river prawns	2017	Philosophical Transactions of the Royal Society of London, Series B: Biological Sciences
Solomons, Noel W	McCollum International Lecturer, Professor Mark L Wahlqvist, points us "Towards a new	2006	The Journal of Nutrition

	generation of international nutritional science and nutritional scientist" from the podium in Durban, South Africa		
Spajic, Luke; Behrens, Georgia; Gralak, Sylvia; Moseley, Genevieve; Linholm, Daniel	Beyond tokenism: meaningful youth engagement in Planetary Health	2019	The Lancet Planetary Health
Stone, Sara B; Almada, Amalia A; Myers, Samuel S	A call for Planetary Health case studies	2017	The Lancet
Stone, Sara B; Myers, Samuel S; Golden, Christopher D; Planetary Health Education Brainstorm Group	Cross-cutting principles for Planetary Health education	2018	The Lancet Planetary Health
Stull, Valerie Jo	Improving global health through insects: The feasibility, acceptability, and potential impact of small-scale mini-livestock farming, with a case study of Zambia	2018	[Doctoral thesis]
Sula-Raxhimi, Enkelejda; Butzbach, Camille; Brousselle, Astrid	Planetary Health: countering commercial and corporate power	2019	The Lancet Planetary Health
Swamy, Latha; Drazen, Erika; Bukoski, Jacob J; Johnson, Whitney R	The future of tropical forests under the United Nations Sustainable Development Goals	2018	Journal of Sustainable Forestry
Tait, Peter	Biosensitivity: the practical pathway to Planetary Health	2018	Australian & New Zealand Journal of Public Health
Taylor, Tim; Mackie, Phil	Carbon footprinting in health systems: one small step towards Planetary Health	2017	The Lancet Planetary Health
The Lancet	Climate change and health - action please, not words	2014	The Lancet
The Lancet	Planetary Health in the Anthropocene	2019	The Lancet
The Lancet Global Health	Vector control: time for a Planetary Health approach	2017	The Lancet Global Health
The Lancet Planetary Health	A sixth mass extinction? Why Planetary Health matters	2017	The Lancet Planetary Health
The Lancet Planetary Health	Promoting Planetary Health through culture	2018	The Lancet Planetary Health
The Lancet Planetary Health	The bigger picture of Planetary Health	2019	The Lancet Planetary Health
The Lancet Planetary Health	Welcome to The Lancet Planetary Health	2017	The Lancet Planetary Health

Veidis, Erika M; Myers, Samuel S; Almada, Amalia A; Golden, Christopher D	A call for clinicians to act on Planetary Health	2019	The Lancet
Velazquez, Jazmin Arguello; Negrutiu, Ioan	Agriculture and global physicochemical deregulation: planetary boundaries that challenge Planetary Health	2019	The Lancet Planetary Health
Vogel, Lauren	Why aren't more doctors talking about climate change?	2019	CMAJ: Canadian Medical Association Journal
Wahl, D. C.	Design for human and Planetary Health: a transdisciplinary approach to sustainability in: Management of Natural Resources, Sustainable Development and Ecological Hazards	2006	WIT Press
Wahlqvist, Mark L.	International trends in nutritionally-related health and their implication for health policy	2008	Asia Pacific Journal of Clinical Nutrition
Walpole, Sarah Catherine; Barna, Stefi; Richardson, Janet; Rother, Hanna-Andrea	Sustainable healthcare education: integrating Planetary Health into clinical education	2019	The Lancet Planetary Health
Watts, Geoff	Judith Rodin: on the path to resilience in Planetary Health	2015	The Lancet
Webb, Kinari	Planetary Health in the tropics: how community health-care doubles as a conservation tool	2018	The Lancet Global Health
Whitmee, Sarah; Haines, Andy; Beyrer, Chris; Boltz, Frederick; Capon, Anthony G; de Souza Dias, Braulio Ferreira; Ezeh, Alex; Frumkin, Howard; Gong, Peng; Head, Peter; Horton, Richard; Mace, Georgina M; Marten, Robert; Myers, Samuel S; Nishtar, Sania; Osofsky, Steven A; Pattanayak, Subhrendu K; Pongsiri, Montira J; Romanelli, Cristina; Soucat, Agnes; Vega, Jeanette; Yach, Derek	Safeguarding human health in the Anthropocene epoch: report of The Rockefeller Foundation-Lancet Commission on Planetary Health	2015	The Lancet

Willett, Walter	Pushing for Planetary Health diets: a summary of the EAT-Lancet Commission report	2019	Food Australia
Wilson, Marisa; Gathorne-Hardy, Alfy; Alexander, Peter; Boden, Lisa	Why "culture" matters for Planetary Health	2018	The Lancet Planetary Health
Wolfson, Mariel Louise	The ecology of a healthy home: energy, health, and housing in America, 1960–1985	2012	[Doctoral thesis]
Xie, Edward; de Barros, Enrique Falceto; Abelsohn, Alan; Stein, Airton Tetelbom; Haines, Andy	Challenges and opportunities in Planetary Health for primary care providers	2018	The Lancet Planetary Health
Yates-Doerr, Emily	Counting bodies? On future engagements with science studies in medical anthropology	2017	Anthropology & Medicine
Yong-Guan Zhu; Gillings, Michael; Simonet, Pascal; Stekel, Dov; Banwart, Steven; Penuelas, Josep	Human dissemination of genes and microorganisms in Earth's critical zone	2018	Global Change Biology
Young, Carrie E.	The impact of narrative and participatory drama on social interactions, attitudes, and efficacy around health and environmental issues in Malawi	2017	[Doctoral thesis]
Zhu, Yong-Guan; Zhao, Yi; Zhu, Dong; Gillings, Michael; Penuelas, Josep; Ok, Yong Sik; Capon, Anthony; Banwart, Steve	Soil biota, antimicrobial resistance and Planetary Health	2019	Environment International
